# Serum Nutrient Levels and Aging Effects on Periodontitis

**DOI:** 10.3390/nu10121986

**Published:** 2018-12-15

**Authors:** Jeffrey L. Ebersole, Joshua Lambert, Heather Bush, Pinar Emecen Huja, Arpita Basu

**Affiliations:** 1 Department of Biomedical Sciences, School of Dental Medicine, University of Nevada Las Vegas, 1001 Shadow Lane, B221, MS 7425, Las Vegas, NV 89106, USA; 2 College of Nursing, University of Cincinnati; Cincinnati, OH 45221, USA; lambejw@ucmail.uc.edu; 3 Department of Biostatistics, College of Public Health, University of Kentucky; Lexington, KY 40536, USA; heather.bush@uky.edu; 4 Department of Periodontics, School of Dentistry, Medical University of South Carolina; Charleston, SC 29425, USA; emecenh@musc.edu; 5 Department of Kinesiology and Nutrition Sciences, School of Allied Health Sciences, University of Nevada Las Vegas, Las Vegas, NV 89106, USA; arpita.basu@unlv.edu

**Keywords:** nutrients, periodontitis, aging

## Abstract

Periodontal disease damages tissues as a result of dysregulated host responses against the chronic bacterial biofilm insult and approximately 50% of US adults >30 years old exhibit periodontitis. The association of five blood nutrients and periodontitis were evaluated due to our previous findings regarding a potential protective effect for these nutrients in periodontal disease derived from the US population sampled as part of the National Health and Nutrition Examination Survey (1999–2004). Data from over 15,000 subjects was analyzed for blood levels of cis-β-carotene, β-cryptoxanthin, folate, vitamin D, and vitamin E, linked with analysis of the presence and severity of periodontitis. Moderate/severe disease patients had lower cis-β-carotene levels across all racial/ethnic groups and these decreased levels in moderate/severe periodontitis were exacerbated with age. β-cryptoxanthin demonstrated lower levels in severe disease patients across the entire age range in all racial/ethnic groups. Folate differences were evident across the various age groups with consistently lower levels in periodontitis patients >30 years and most pronounced in females. Lower levels of vitamin D were consistently noted across the entire age range of patients with a greater difference seen in females with periodontitis. Finally, an analytical approach to identify interactions among these nutrients related to age and periodontitis showed interactions of vitamin D in females, and folate with race in the population. These findings suggest that improving specific nutrient intake leading to elevated blood levels of a combination of these protective factors may provide a novel strategy to affect the significant increase in periodontitis that occurs with aging.

## 1. Introduction

Periodontitis and dental caries remain the two major oral health maladies across the lifespan in the United States [1]. The National Health and Nutrition Examination Survey (NHANES) (2009–2012) reported that approximately 50% of US adults greater than 30 years old exhibit periodontitis, with minorities and older individuals being disproportionately affected [2]. Based on the Global Burden of Disease 2010 study, severe periodontitis is a significant health burden, affecting approximately 743 million people [3]. Periodontal disease damages tissues as a result of dysregulated host responses against the chronic bacterial biofilm insult [4,5,6]. It represents the primary basis of adult tooth loss, substantially affecting the individual’s quality of life [7,8,9]. Similar to many chronic diseases, periodontal disease is a complex disease driven by genetic and epigenetic influences, patient behaviors, medication use, and/or environmental factors that promote periodontal disease initiation and progression [10]. Moreover, smoking is clearly one of the most significant modifiable risk factors in the pathogenesis of periodontitis and disease extent and severity [11,12].

Considerable effort has occurred in attempting to define various genetic influences on the population-based extent and severity of the disease [13,14,15,16]. These studies have identified some unique subpopulations with clearly altered host functions that increase susceptibility, based on various single nucleotide polymorphisms (SNP) or mutations including genes controlling the production of inflammatory mediators and tissue and bone regulatory molecules [17,18,19,20,21,22], as well as some genome-wide association studies (GWAS) that have attempted to identify gene(s) that confer risk for disease [14,19,23,24,25]. More recent reports have also described epigenetic alterations in the genomes of periodontitis patients [26,27,28,29,30,31,32]. However, with complex diseases, such as periodontitis, there are likely a plethora of critical gene–environment interactions that occur to alter individual risk and enable gene effects to help explain disease phenotypes.

Environmental epidemiology focuses on the discovery of environmental exposures that contribute to or protect against diseases and the identification of public health actions to effectively manage the risks associated with harmful exposures. Environmental exposures can be involuntary or represent occupational exposures and voluntary exposures such as smoking, medications, and diet. While periodontitis is a localized inflammatory process mediating destruction of soft and hard periodontal tissues [33] resulting from the chronic disruption of the oral epithelial barrier, this disease triggers systemic inflammatory host responses that may contribute to other systemic conditions (e.g., cardiovascular disease, diabetes, Alzheimer’s disease [34,35,36,37]). Additionally, the systemic contribution to the microenvironment in periodontal tissues is a critical component for maintaining homeostasis or influencing the progression of disease [38]. This breadth of data identifies the need for optimal oral health as an integral component of the prevention and management of chronic health conditions.

Observational studies have shown a strong correlation between the intake of fruits and vegetables and other antioxidant nutrients, with oral health-related quality of life in adults [39,40,41,42]. These effects have been mainly attributed to their function in reducing oxidative stress and inflammation, and were related to intake of β-carotene, vitamin C, a-tocopherol, and omega-3-fatty acids [33,43,44]. However, while dietary nutrients and specific bioactive compounds have emerged as influential factors in the etiology and progression of periodontitis [45], a recent systematic review [46] emphasized associations, but also the general lack of clear data supporting causal relationships.

We have reported on an Environment-Wide Association Study of periodontitis in data derived from the National Health and Nutrition Examination Survey from 1999–2004 [47]. These findings identified a number of environmental toxins/factors that significantly increased the prevalence of periodontitis, particularly related to age, race/ethnicity, and smoking status. However, we also noted a number of serum nutrients that were significantly negatively associated with the prevalence, including cis-β-carotene, vitamin D, vitamin E, folate, and β-cryptoxanthin. These findings are consistent with recent NHANES reports on cis-β-carotene and peripheral artery disease [48] and for cis-β-carotene, vitamin D, vitamin E, and β-cryptoxanthin coupled with physical activity in lowering the risk for metabolic syndrome [49]. Linden et al. [50] also reported elevated levels of cis-β-carotene and β-cryptoxanthin in males from 60–70 years of age associated with decreased periodontitis.

While these reports are promising, further research deserves urgent attention given the high prevalence of periodontitis in the US and global population. Based on the results from various experimental models and clinical studies, designing dietary or supplemental formulations may present as an emerging line of natural therapy for periodontitis and to maximize population levels of oral health.

## 2. Materials and Methods

### 2.1. Population Data

In this study, periodontal examination data from public-use data files for three NHANES cohorts—1999–2000, 2001–2002, 2003–2004—were extracted and combined to comprise the study population. These surveys, using the same methods, assessed the health status of a nationally representative sample of the civilian non-institutionalized US population, selected through a stratified multistage probability sampling design. Full descriptions of the sample design for these NHANES datasets are publically available [51]. NHANES 1999–2000 (N = 9956), 2001–2002 (N = 10,477), and 2003–2004 (N = 9643) enlisted persons 1 month of age or older. Among the 11,837 participants who were equal to and older than 18 years of age and had 16 or more teeth, 3745 were collected in the first cohort (1999–2000), 4258 in the second cohort (2001–2002), and 3834 in the third cohort (2003–2004). Those with missing smoking status and periodontal parameters were excluded leaving a final analytical sample of 8884 participants (1999–2000: 2738; 2001–2002: 3294; 2003–2004: 2852). The NHANES is a complex, multistage probability sample of non-institutionalized US civilians and subsequently organized into six unique datasets derived from 2-year cycle population sampling (Centers for Disease Control and Prevention; National Center for Health Statistics). Each 2-year survey cycle examines a representative US sample of approximately 10,000 persons and collects health-related data.

### 2.2. Demographics

The demographic variables considered in this study included age, gender, race, socio-economic status, smoking status, and number of teeth. Racial-ethnic groups were summarized into five categories: Mexican American, other Hispanic, non-Hispanic white, non-Hispanic black, and other race, as shown in Table S1. Socio-economic status, estimated using the poverty income ratio, was computed as the ratio of family/individual income to the appropriate federal poverty threshold. Smoking status—current smoker, former smoker, non-smoker—was derived from the two self-reported questions. Participants reported having historically smoked more than 100 cigarettes, but currently not smoking were defined as former smokers. Non-smokers were defined as reporting never smoking.

### 2.3. Clinical Parameters

The analysis included only the records of adults 18 years or older who had a periodontal examination during the NHANES 1999–2000 (N = 5206), 2001–2002 (N = 5587), and 2003–2004 (N = 5051). Thus, the combined sample for clinical data was 15,844.

Dentists trained in the survey examination protocol conducted the periodontal examinations collecting probing depth and attachment loss measurements [52,53,54]. Periodontitis was defined as a site with clinical attachment loss (CAL) ≥3 mm and a periodontal pocket ≥4 mm. NHANES (1999–2004) used the partial-mouth periodontal examination (PMPE) protocol to sample teeth and sites. The PMPE protocols randomly selected two quadrants of the mouth and specified two to three sites per tooth for measurement of pocket depth, attachment loss, and bleed on probing. In 1999–2000, two sites per tooth (mid-facial and mesio-facial) were measured, while three sites per tooth (mid-facial, mesio-facial, and distal) were measured in 2001–2002 and 2003–2004. A periodontal site was defined as a pocket site with ≥3 mm CAL and ≥4 mm PD (Health and Human Services Vital and Health Statistics Series 11 Report). The gold standard of diagnosing periodontitis using full-mouth examination protocol yields an approximated prevalence of 22.4% [55,56]. The half-mouth (16 teeth) examination protocol utilized by NHANES is known to underestimate the national prevalence rate since periodontitis is site-specific and not evenly distributed in the mouth [55]. Thus, case definition of Series 11 was chosen because it yielded an estimate (16.9%) in NHANES 2001–2004 that was closest to the gold standard. The level of periodontitis—mild, moderate, and severe—was defined as described by Page and Eke [56].

### 2.4. Environmental Variables

The environmental factors were categorized into 15 classes based on NHANES categorization. Environmental variables measured in at least one of the three data cohorts (i.e., 1999–2004) were included in the study. A total of 156 environmental factors were measured in the NHANES data using blood and urine samples. These included chemical toxicants, pollutants, allergens, bacterial/viral organisms, and nutrients. Within the nutrient category there were 16 measures, as shown in Table S2. Factors with laboratory measurements that had greater than 10% of the observations below a detection limit threshold defined by NHANES were omitted from analysis. The laboratory measurements using mass spectrometry and absorption spectroscopy demonstrated that the majority of the variables were detected in small ranges and were skewed. Thus, all environmental variables were log-transformed (natural) and standardized and referred to as “processed”. Levels presented were back-transformed from log-scale values.

### 2.5. Statistical Approaches

Descriptive statistics were calculated for each serum nutrient, and data are presented as means and one standard deviation, and stratified by severity of periodontitis, race, and smoking status. Survey-weighted logistic regressions were performed for each of the processed environmental factors, adjusting for age, gender, ethnicity, socio-economic status, smoking status, and number of teeth. The R package “survey” was used in R (Version 3.1.2) for the survey-weighted logistic regression [57]. Weights were constructed in SAS (Version 9.4, Cary, NC, USA) using a 6 year weighting design from the NHANES variable WTMEC2YR73 [58]. Adjusted odds ratios were calculated with 95% confidence intervals and were provided to demonstrate the association between the individual factors and periodontitis. These regressions were repeated by smoking status to examine potential associations within smoking categories.

Using survey-weighted logistic regressions, subgroup specific effects were explored using the feasible solution algorithm (FSA) [59]. The FSA can be employed to identify complex subgroup specific effects in datasets with many variables (such as the NHANES dataset). Subgroup specific effects were identified using FSA by checking for the statistical significance of two-way interactions. The FSA is very flexible in the interactions it seeks to identify and can limit the variables it uses in the procedure. In these analyses, we sought to identify nutrient interactions between age, sex, or race/ethnicity. Because this process was exploratory in nature, interactions were searched for with the adjustment of other known covariates or confounders. The odds ratios for the interactions identified are reported as well as their 95% confidence intervals.

## 3. Results

Of the nutrients examined in the NHANES dataset, five demonstrated a significant relationship to the prevalence of periodontitis across the various demographic cohorts; cis-β-carotene, vitamin D, vitamin E, folate, and β-cryptoxanthin.

**Figure 1:**
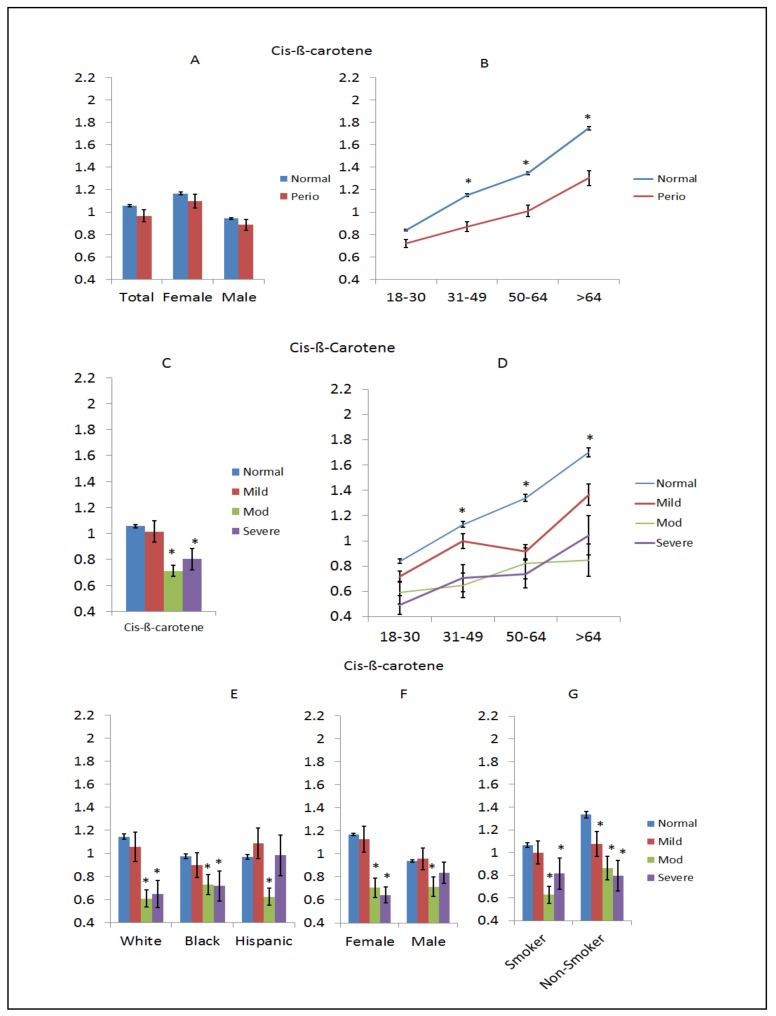
Levels of cis-β-carotene (µg/dL) in subjects with or without periodontitis stratified upon sex (**A**), age (**B**), disease severity (**C**), disease severity and age (**D**), race/ethnicity (**E**), disease severity and sex (**F**), and disease severity in smokers (**G**). The bars denote group means and the vertical brackets enclose 1 standared error of the mena (SEM). The asterisk (*) denotes significantly different than the normal group at *p* < 0.05.

Cis-β-carotene is a tetraterpene composed of two retinyl groups, and is broken down in the mucosa of the human small intestine to retinal, a form of vitamin A. The results in Figure 1A–G summarize the distribution of cis-β-carotene levels in serum across the population. Figure 1A shows some decreased level in periodontitis in examining the entire population, which were reflected in both sexes; however, Figure 1B emphasizes the lower level of this dietary nutrient that becomes more pronounced at >30 years of age in the patients with periodontitis. Figure 1C explores the levels related to the severity of periodontitis and shows decreased levels in moderate and severe periodontitis, which is exacerbated with age, as shown in Figure 1D. Interestingly, even the older mild periodontitis patients displayed markedly lower level of cis-β-carotene. Based upon previous data supporting race/ethnicity and smoking as factors in the expression of periodontitis, Figure 1E demonstrates that severe and/or moderate disease patients had lower cis-β-carotene levels across all the racial/ethnic groups. Examination of severity of disease stratified by sex of cis-β-carotene levels is shown in Figure 1F, demonstrating lowered levels of this nutrient in blood of both sexes with more advanced disease. Also, while moderate and severe disease smokers had lower levels of this nutrient, in the non-smokers there was a continuous decrease across all categories of periodontitis versus the subjects with a healthy periodontium, as shown in Figure 1G.

**Figure 2:**
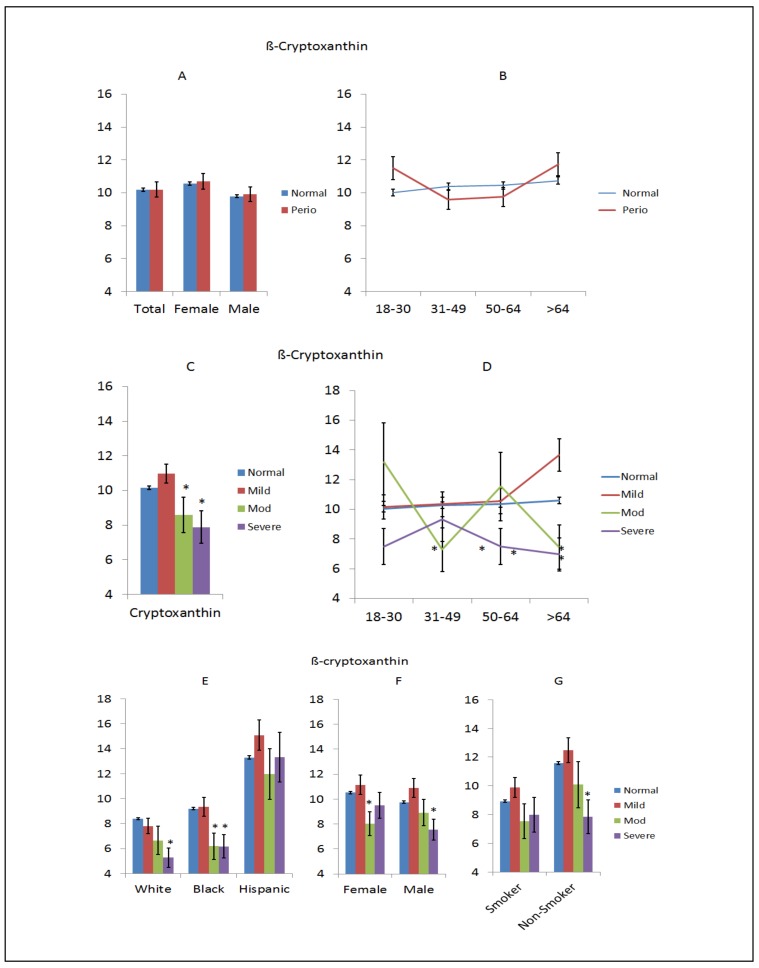
Levels of β-cryptoxanthin in subjects with or without periodontitis stratified upon sex (**A**), age (**B**), disease severity (**C**), disease severity and age (**D**), race/ethnicity (**E**), disease severity and sex (**F**), and disease severity in smokers (**G**). The bars denote group means and the vertical brackets enclose 1 SEM. The asterisk (*) denotes significantly different that other group(s) at *p* < 0.05.

β-Cryptoxanthin is a member of the class of carotenoids known as xanthophylls and is found in fruits and vegetables. It is closely related to β-carotene, and can be converted to vitamin A although not as effectively as the di-retinyl rings of β-carotene. As with other carotenoids, it is an antioxidant and appears to be associated with decreased risk of some cancers, degenerative diseases, and may positively affect bone in osteoporosis. Figure 2 provides an overview of the relationship of this dietary nutrient to periodontitis. Figure 2A shows no difference in levels between normal (non-periodontitis) and periodontitis across the entire population or based on sex, nor was there a noticeable effect of aging on the levels between these 2 groups, as shown in Figure 2B. However, Figure 2C shows decreased levels in moderate and severe periodontitis patients compared to normal (non-periodontitis) subjects, and Figure 2D demonstrates lower levels in severe disease patients across the entire age range. Figure 2E shows lower levels of this nutrient in more advanced diseases in white and black racial/ethnic groups, although there was no effect and a dramatically higher level of β-cryptoxanthin in Hispanic subjects. Similar decreased levels were found in moderate-severe disease in both sexes and in smokers and non-smokers, as shown in Figure 2F,G.

**Figure 3:**
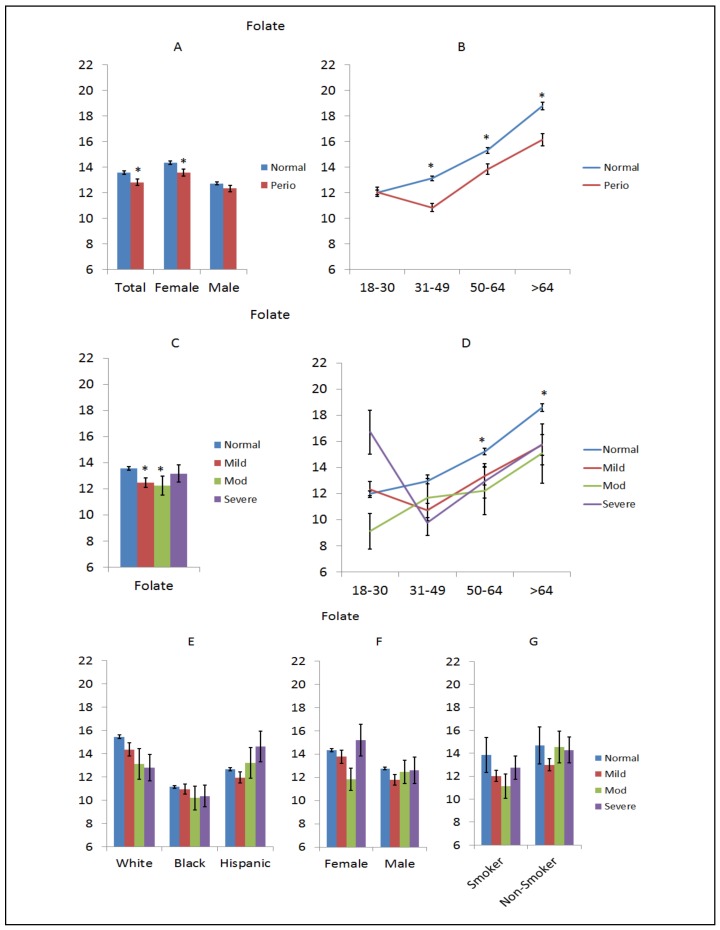
Levels of folate (g/mL) in subjects with or without periodontitis stratified upon sex (**A**), age (**B**), disease severity (**C**), disease severity and age (**D**), race/ethnicity (**E**), disease severity and sex (**F**), and disease severity in smokers (**G**). The bars denote group means and the vertical brackets enclose 1 SEM. The asterisk (*) denotes significantly different than the normal group at *p* < 0.05.

Folate is one of the B vitamins, vitamin B9, which occurs naturally in many foods, especially dark green leafy vegetables. Figure 3 summarizes the distribution of folate levels in the NHANES population, as related to periodontal disease. For the overall population, levels of folate were decreased in the periodontitis patients and this difference was most pronounced in female subjects, as shown in Figure 3A. This difference was even more evident across the various age groups with consistently lower levels in periodontitis patients >30 years of age, as shown in Figure 3B. Stratified based on disease severity, small but significant differences were observed in mild and moderate periodontitis subsets, as shown in Figure 3C. The decreased levels of folate were accentuated in patients >30 years of age, as shown in Figure 3D. A different pattern of folate levels was noted among the racial/ethnic groups, with decreasing levels with disease severity in whites and increasing levels in disease severity in Hispanics. Generally similar levels were seen in the black subjects, as shown in Figure 3E. Related to disease severity, no consistent differences were seen based upon sex, as shown in Figure 3F. In smokers, lower folate levels were associated with all levels of periodontitis, although this was not seen in the non-smoking subgroup, as shown in Figure 3G.

**Figure 4:**
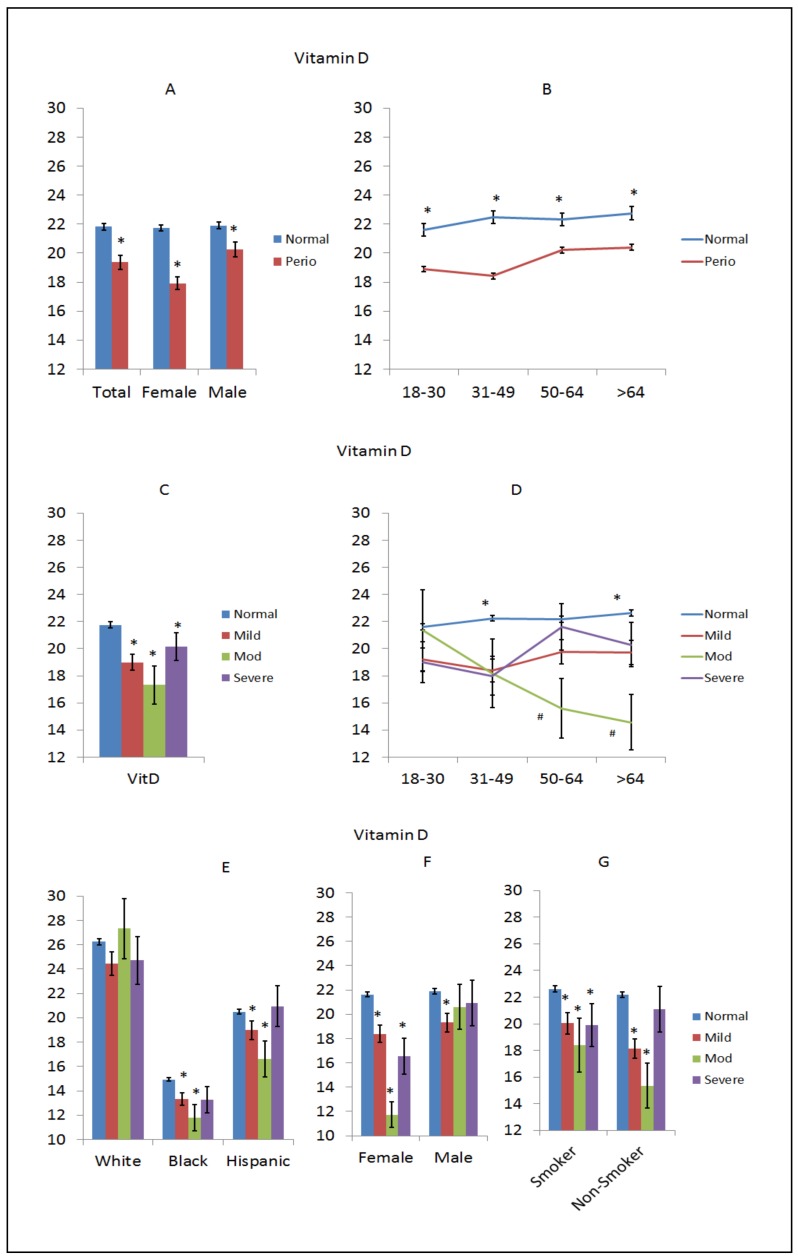
Levels of vitamin D (ng/mL) in subjects with or without periodontitis stratified upon sex (**A**), age (**B**), disease severity (**C**), disease severity and age (**D**), race/ethnicity (**E**), disease severity and sex (**F**), and disease severity in smokers (**G**). The bars denote group means and the vertical brackets enclose 1 SEM. The asterisk (*) denotes significantly different than the normal group at *p* < 0.05.

Vitamin D is technically a group of fat-soluble hormones that impact intestinal absorption of calcium, magnesium, and phosphate. In particular, vitamin D3 and D2 can be ingested from the diet, with the major natural source of this vitamin being synthesis from cholesterol in the skin via sun exposure. Vitamin D is hydroxylated to form calcitriol, which has a major role regulating the concentration of calcium and phosphate for bone growth and remodeling. Recently, calcitriol has also been identified to have important functions in regulating immune and inflammatory responses [60]. Lower levels of vitamin D were seen in the periodontitis population and in both sexes, with a greater difference in females with periodontitis compared to normal (non-periodontitis) females, as shown in Figure 4A. Lower levels were consistently noted across the entire age range of patients with rather minimal variation with aging, as shown in Figure 4B. The lowest levels of vitamin D were noted in patients with moderate periodontitis, particularly in patients over 50 years of age, as shown in Figure 4C,D. Figure 4E shows exceptionally low vitamin D levels in all black subjects with decreased levels in both black and Hispanics with moderate periodontitis. There was a decreased level of vitamin D in females with all levels of periodontitis and a nearly 2-fold decrease in females with moderate periodontitis, as shown in Figure 4F, with no differences seen in males. Smokers did not appear to have decreased vitamin D levels, although disease in both smokers and non-smokers was generally associated with lower vitamin D levels, as shown in Figure 4G.

**Figure 5:**
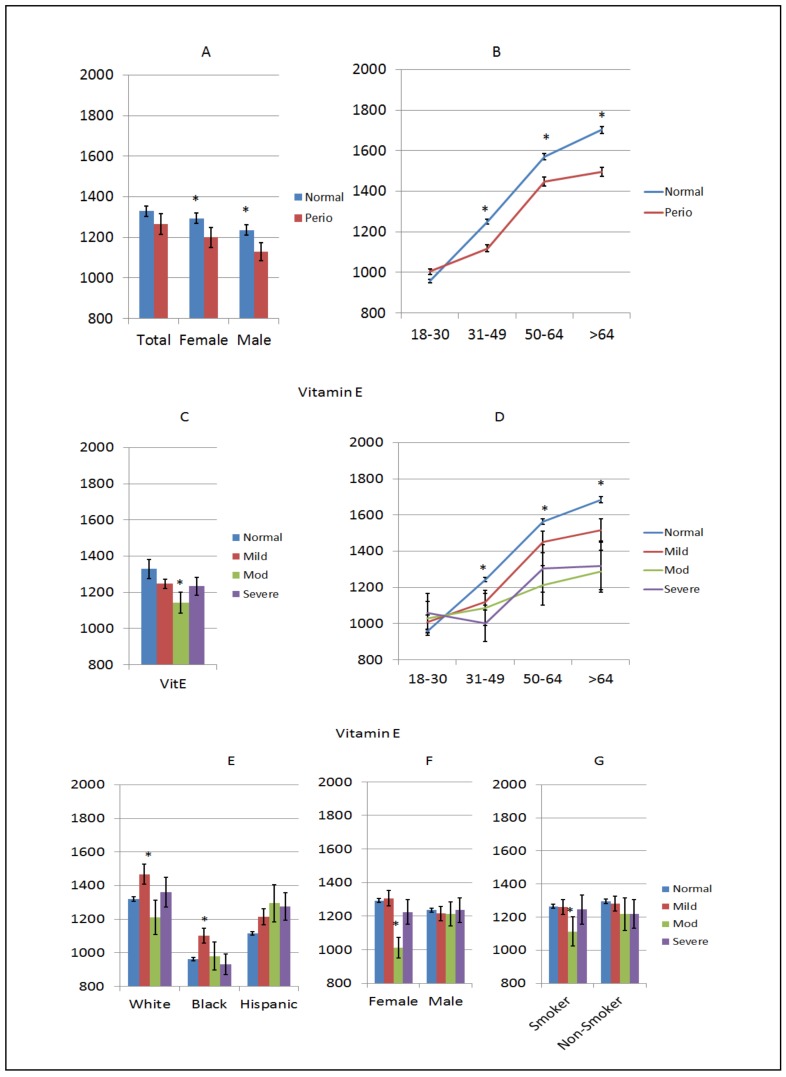
Levels of vitamin E (µg/dL) in subjects with or without periodontitis stratified upon sex (**A**), age (**B**), disease severity (**C**), disease severity and age (**D**), race/ethnicity (**E**), disease severity and sex (**F**), and disease severity in smokers (**G**). The bars denote group means and the vertical brackets enclose 1 SEM. The asterisk (*) denotes significantly different than the normal group at *p* < 0.05.

Vitamin E is a group of eight fat-soluble compounds including tocopherols and tocotrienols that can act as antioxidants and easily penetrate cell membranes. Levels of vitamin E were increased in both normal males and females compared to periodontitis, as shown in Figure 5A. This was particularly noted with lower levels observed in periodontitis patients >30 years of age, and the largest difference from normal subjects was seen in the oldest disease patients, as shown in Figure 5B. While all levels of periodontitis showed lower vitamin E levels, only the moderate disease group reached statistical significance, as shown in Figure 5C. As seen in the overall population, patients >30 years of age showed lower levels of vitamin E related to periodontitis severity and age compared to normal subjects, as shown in Figure 5D. Generally, disease patterns in vitamin E levels were not noted based on race/ethnicity, albeit the black subjects routinely had the lowest levels of vitamin E, as shown in Figure 5E. Based upon the severity of the disease, males showed no differences in vitamin E levels and while females with moderate disease had decreased blood levels of vitamin E, there was no particular pattern of alterations related to disease versus normal subjects, as shown in Figure 5F. Similarly, rather minimal differences were observed when subjects were classified based upon smoking, as shown in Figure 5G.

The previous results presented the five nutrients individually related to disease and various demographic characteristics. However, the functional activity of these nutrients would be expected to be acting in concert, with potential additive or synergistic contributions to the overall susceptibility or resistance to periodontal disease initiation and progression. Thus, we employed the FSA to estimate the varying effects of blood nutrient levels within age, race/ethnicity, and sex that are significantly associated with periodontal health. Two significant and near significant interactions were found from the FSA analysis. Vitamin D and folate were found to have varied effects for different subgroups of sex, age, and race, respectively. All reported interaction odds ratios were adjusted for the main effect of age, ratio of family income to poverty, sex, and race and interpreted using a log standardized scale. One additional log standardized unit of vitamin D in females had an estimated adjusted odds of periodontal disease 0.86 (95% CI: 0.71, 1.03; *p*-value = 0.124) times that of males. One additional log standardized unit of folate in whites had an estimated adjusted odds of periodontal disease 0.78 (95% CI: 0.61, 1.01; *p*-value = 0.059) times that of Hispanics.

## 4. Discussion

Periodontitis is considered a dysregulation of host responses to an evolving dysbiotic microbiome at sites of lesions reflecting a chronic local inflammatory environment [36,61]. While data derived from technologies developed for the Human Microbiome Project [62] to profile the members and functions of the bacteria in health and pathogenic biofilms [63] has provided new insights into this microbial dysbiosis, there appears a clear role for individual genetic variation across the population that contributes to disease expression and severity [17,19,24,26,30,64,65,66]. Furthermore, aging and race/ethnicity increase the risk for the extent/severity of periodontitis [67], and more recently, gene-environment interactions contributing to disease have emphasized the importance of the exposome in affecting disease risk [27,29,68].

In a previous study of the exposome using NHANES data from 1999–2004, we identified more classical factors (i.e., age, gender, race/ethnicity) in disease prevalence, and for the first time incorporated a broad array of environmental variables that significantly enhanced the prevalence of periodontitis in the population [47]. However, we also noted a select group of blood nutrients that demonstrated a significant protective odds ratio for periodontitis that showed an increased affect with aging.

This exploratory report provides additional details on five of the blood nutrients—cis-β-carotene, folate, vitamin D, vitamin E, and β-cryptoxanthin—that demonstrated these protective associations. There are over 600 known carotenoids: [xanthophylls (β-cryptoxanthin, lutein, zeaxanthin; non-vitamin A carotenoids) and carotenes (β-carotene, α-carotene, lycopene)]. Generally, the health benefits of carotenoids are considered to interact with endogenous antioxidant enzymes to positively affect inflammation and immune responses [69]. Carotenoids have been shown to alter intracellular inflammatory signaling pathways (e.g., NFκB) and inflammatory mediator profiles [70]. These effects have been evaluated in various studies related to periodontal disease. Periodontitis leads to significant increases in an array of systemic acute phase proteins, with the literature supporting increased systemic inflammation with low vitamin A levels [71,72]. Our data demonstrated a significantly decreased level in cis-β-carotene in the serum of periodontitis patients with a greater divergence—in the subjects with a normal periodontium and advancing age. This is consistent with a report of an inverse relationship between elevated carotenoids and serum C-reactive protein (CRP) levels in 60–70 year old men [73], as well as data supporting a link between dietary carotenoids and cognitive functions in humans [74]. We noted this difference from blood levels in subjects with a normal periodontium that was accentuated with more severe disease in the aging population. Our data also showed this relationship with more severe disease across racial/ethnic groups, particularly in white and black subjects, and in both smokers and non-smokers. These effects have been evaluated in a limited number of studies related to periodontal disease. Low blood levels of various carotenoids were correlated with an increased prevalence of periodontitis in older men [50]. Moreover, in non-smokers, serum carotenoid levels interacted with clinical improvement in periodontitis following scaling and root planing [39]. Our findings from the large NHANES cohort supports these findings and suggested that increased dietary availability of carotenoids could contribute to a treatment strategy for increasing prevalence of periodontitis with aging.

Dietary β-cryptoxanthin, another carotenoid, also displays anti-inflammatory activities that positively affect various chronic inflammatory diseases including polyarthritis [75] and osteoarthritis [76]. Data from studies of inflammation in metabolic syndrome supported an effect on regulating NF-κB and Nrf2 pathways that control inflammatory mediators and antioxidant proteins [77]. Findings from Matsumoto et al. [78] found that β-cryptoxanthin suppressed lipopolysaccharide (LPS)-induced osteoclast formation and lowered alveolar bone loss in a mouse model and decreased *Porphyromonas gingivalis*-induced IL-6 and IL-8 production by human periodontal ligament cells [79]. As noted by Toti et al. [80], this type of dietary immunomodulatory could be considered part of a personalized nutritional or supplementation strategy for preventing and treating chronic inflammatory conditions, such as periodontitis.

Folate is one of the B vitamins found mainly in dark green leafy vegetables, beans, peas and nuts, and fruits (oranges, lemons, bananas, melons, and strawberries). Humans cannot produce folic acid making it an essential nutrient required from the diet and critical for synthesis of DNA, RNA, and metabolizing amino acids. While folate has been most directly linked to pregnancy and preventing neural tube defects and a type of anemia, supplementation has been associated with some reductions in the risk of cardiovascular disease [81]. Folic acid has also been shown to regulate inflammation driven by release of endogenous danger-associated molecular pattern (DAMP) molecules [82], as well as regulating reactive oxygen species production during hypoxia [83]. However, a recent clinical trial did not identify an impact on systemic inflammation and endothelial dysfunction in women [84]. Previous findings have reported lower folic acid levels in smokers with periodontal disease [85] and that elevated folate (B-complex) levels provided a positive impact on nonsurgical periodontal therapy and periodontal wound healing [86]. However, these same authors suggested a general lack of data to support an impact of nutritional supplementation, including B-complex, on preventing periodontal disease [87]. Staudte et al. [88] did demonstrate that a lower intake of dietary folic acid was related to an increased prevalence of periodontitis. Our results demonstrated lower folate levels with periodontitis, particularly in subjects >30 years old, which was reflected in patterns for all levels of disease extent. Additionally, lower folate levels paralleled disease extent in white subjects and in smokers, with an opposite presentation in Hispanic subjects that is not readily explained from the existing literature of folate levels in this ethnic group. Interestingly, our findings with folate, using somewhat different clinical definitions on a broader NHANES population base, reflected the results of Yu et al. [89] who reported that low folate levels were an independent risk factor for periodontal disease in older adults. Thus, while this exploratory study only provides associational outcomes, this dietary nutrient may contribute as a clinical target for prevention or early intervention in effectively managing periodontal health, particularly in the aging population.

Numerous reports have linked increased levels of vitamin D with minimizing tooth loss resulting from periodontitis [90,91,92,93,94,95,96]. Additionally, vitamin D receptor polymorphisms [97] and for vitamin D binding protein appear to have some impact on increasing the risk for periodontitis [98,99]. Research has also found that patients with low serum vitamin D levels, exhibited healing from periodontal surgery was adversely affected [100]. Related to these clinical findings, vitamin D improves epithelial innate immune functions and antimicrobial peptide secretion [101,102,103] and down-regulates NFκB activation and cytokine secretion by monocytes and macrophages [104,105]. Additionally, from the microbiome side of the periodontal disease equation, vitamin D decreases virulence gene expression by *P. gingivalis* [104,105]. Our analysis of this nutrient based upon examination of NHANES data demonstrated lower blood levels in periodontitis patients across the entire age range, suggesting the potential for a protective relationship. This difference was exacerbated in older individuals with moderate periodontitis, and particularly in black and Hispanic subjects. This was an unexpected finding as one might hypothesize a dose effect, whereby this type of protective nutrient would be more greatly affected in severe periodontitis patients. Potentially, those patients with moderate disease are most affected by alterations in this nutrient, while the mild disease individuals with lower levels exhibited a relationship with periodontitis that was more variable in the heterogeneous population. Moreover, with the severe disease patients, existing literature supports that this might be a unique subset of patients with multiple risk factors (e.g., genetics, microbiome) increasing the risk of severity of disease [67,68,69,70,71,72,73,74,75,76,77,78,79,80,81,82,83,84,85,86,87,88,89,90,91,92,93,94,95,96,97,98,99,100,101,102,103,104,105,106] that may simply overwhelm any impact of the vitamin D effects. The effect of vitamin D was also observed in both smokers and non-smokers. This relationship of vitamin D levels and general health has also been reported using NHANES data [107,108,109], suggesting an important role for enhancing the proportion of the population of all age groups with normal levels of vitamin D.

The tocopherols and tocotrienols occur in α, β, γ, and δ forms with γ-tocopherol the most common form of vitamin E found in the North American diet and is the major tocopherol in corn oil and soybean oil, while α-tocopherol is the most biologically active form of vitamin E. These are fat-soluble antioxidants that help block the formation of reactive oxygen species by interacting with cellular membranes and adipose tissues. An increased consumption of vitamin E has been related to a lower incidence of cardiovascular disease (CVD), cancer and dementia, although data from randomized trials did not consistently demonstrate an effect with these diseases [110,111,112,113]. The literature with vitamin E in periodontitis is somewhat limited. Patients with serum α-tocopherol levels that were in the low normal range displayed an increased severity of periodontitis [114]. Dietary or supplements with vitamin E showed a positive impact on clinical measures of chronic periodontitis, potentially through their effect on oxidative stress reactions to chronic inflammation [115]. A study of scaling and root planing with vitamin E supplementation appeared to improve periodontal wound healing and improved serum and salivary superoxide dismutase activities [116]. Moreover, increased dietary intake of α-tocopherol with other antioxidants and anti-inflammatory omega-3 fatty acids reduced probing depths in chronic periodontitis that was limited to only non-smokers [39]. In contrast, while Linden et al. [50] found an effect of carotenoids on periodontitis in older men, serum vitamin E levels were unrelated to disease. Mechanistically, α-tocopherol has been shown to modulate the production of reactive oxygen species by neutrophils following activation by FcγR and TLR ligands [117]. This form of vitamin E also decreases gingival fibroblast production of IL-1β and IL-6, while increasing various human β-defensins following challenge with LPS from *P. gingivalis* [118]. Thus, numerous studies support the potential that optimal vitamin E levels may contribute to an overall improved host anti-inflammatory environment, particularly in concert with a panel of dietary nutrients directed toward controlling oxidative stress and associated chronic inflammatory reactions.

This report describes an associational study of a large US population sampled over an interval of 5 years via the NHANES project and demonstrated statistical associations of a subset of serum nutrients to the expression of periodontitis. The concept of diet impacting periodontal disease has been the subject of a number of reports [45,46] that can be implemented via specific strategies related to food intake [39,88] and specific supplementation [119]. While these studies have often focused on the capacity of these nutrients to act as antioxidants cooperating with endogenous host antioxidant biomolecules [40], an important concept that emerges is a lower likelihood of a single nutrient provided through food or supplements as being optimally effective in the prevention or treatment of inflammation and alveolar bone loss in periodontitis. However, targeted combinations of these biologically active nutrients may provide a solid strategic adjunctive approach to the management of periodontitis, particularly with aging. Our results indicated interactions between vitamin D and sex, and race and folate. The estimated effect of vitamin D was shown to be more protective in females than males. Moreover, the estimated protective effect of folate was greater in whites than it was in Hispanics.

As with all cross-sectional observational epidemiologic studies, this evaluation cannot define a cause and effect relationship between the functions of the nutrients and disease outcomes. Moreover, it is well recognized that the NHANES clinical design did not provide the detailed clinical evaluation of periodontitis that represents current concepts of disease expression and progression. However, the findings related to the potential impact on disease in aging, associated with race/ethnicity as a risk for disease, and smoking effects, suggest that the nutrient components could be evaluated in more prospective studies, to delineate causal linkages in protection from expression of periodontitis. Delivery of an optimized combination of these nutrients at each meal or via snacks, in combination with adequate measures of standard oral hygiene care, may provide evidence for an important role in the prevention of periodontitis.

## Supplementary Materials

The following are available online at http://www.mdpi.com/2072-6643/10/12/1986/s1, Table S1: Population demographics, Table S2: Serum nutrient variables.
