# Supplementary material for: Serum Nutrient Levels and Aging Effects on Periodontitis
Source: Nutrients. 2018 Dec 15;10(12):1986. doi: 10.3390/nu10121986 (PMC6316450; doi:10.3390/nu10121986)
Supplement: Supplementary file 1 [file nutrients-10-01986-s001.pdf]

Supplementary Table 1: Population demographics.

			Periodontitis		
**Group**	**Nutrient**	**Normal**	**Mild**	**Moderate**	**Severe**
**Race/Ethnicity**		**N**	**N**	**N**	**N**
White	LBXVID	4274	91	9	24
Black	LBXVID	2216	89	14	19
Hispanic	LBXVID	2818	111	13	18
White	LBXVIE	5572	122	14	40
Black	LBXVIE	2788	135	26	24
Hispanic	LBXVIE	4024	187	22	44
White	LBXCBC	4266	90	9	24
Black	LBXCBC	2224	89	14	19
Hispanic	LBXCBC	2828	111	13	18
White	LBXFOL	5626	123	14	41
Black	LBXFOL	2811	137	26	24
Hispanic	LBXFOL	4044	187	22	44
White	LBXCRY	4247	90	9	24
Black	LBXCRY	2222	88	14	19
Hispanic	LBXCRY	2824	111	13	18
**Age**					
18-30	LBXVID	2563	27	3	2
31-49	LBXVID	2253	144	22	18
50-64	LBXVID	1230	67	5	26
above 65	LBXVID	1132	58	7	17
18-30	LBXVIE	3704	57	5	2
31-49	LBXVIE	3247	208	33	26
50-64	LBXVIE	1792	104	15	49
above 65	LBXVIE	1624	85	11	34
18-30	LBXCBC	2570	27	3	2
31-49	LBXCBC	2258	144	22	18
50-64	LBXCBC	1233	66	5	26
above 65	LBXCBC	1131	58	7	17
18-30	LBXFOL	3730	57	5	2
31-49	LBXFOL	3274	209	33	26
50-64	LBXFOL	1804	106	15	49
above 65	LBXFOL	1641	85	11	35
18-30	LBXCRY	2564	27	3	2
31-49	LBXCRY	2250	143	22	18
50-64	LBXCRY	1228	66	5	26
above 65	LBXCRY	1129	58	7	17

Supplementary Table 2: Serum nutrient variables.

Variable ID	Variable	Concentration
LBXFOL	Folate	g/mL
LBXRPL	Retinyl palmitate	μg/dL
LBXVIA	Vitamin A	μg/dL
LBXB12	Vitamin B12	pg/mL
LBXVIE	Vitamin E	μg/dL
LBXVID	Vitamin D	ng/mL

LBXVIA	Retinol	μg/dL
LBXVIE	alpha-tocopherol	μg/dL
LBXGTC	gamma-tocopherol	μg/dL
LBXALC	alpha-carotene	μg/dL
LBXBEC	trans-beta-carotene	μg/dL
LBXCBC	cis-beta-carotene	μg/dL
LBXCRY	beta-cryptoxanthin	μg/dL
LBXLUZ	Combined Lutein/zeaxanthin	μg/dL
LBX2LYC	trans-lycopene	μg/dL
